# The Protein Kinase Double-Stranded RNA-Dependent (PKR) Enhances Protection against Disease Cause by a Non-Viral Pathogen

**DOI:** 10.1371/journal.ppat.1003557

**Published:** 2013-08-22

**Authors:** Pauline Sebby Ogolla, Jose-Andres C. Portillo, Christine L. White, Krupen Patel, Bruce Lamb, Ganes C. Sen, Carlos S. Subauste

**Affiliations:** 1 Department of Pathology, Case Western Reserve University, Cleveland, Ohio, United States of America; 2 Division of Infectious Diseases and HIV Medicine, Department of Medicine, Case Western Reserve University School of Medicine, Cleveland, Ohio, United States of America; 3 Department of Molecular Genetics, Lerner Research Institute, Cleveland Clinic, Cleveland, Ohio, United States of America; 4 Department of Neurosciences, Lerner Research Institute, Cleveland Clinic, Cleveland, Ohio, United States of America; 5 Department of Ophthalmology and Visual Sciences, Case Western Reserve University, Cleveland, Ohio, United States of America; University of Medicine and Dentistry of New Jersey, United States of America

## Abstract

PKR is well characterized for its function in antiviral immunity. Using *Toxoplasma gondii*, we examined if PKR promotes resistance to disease caused by a non-viral pathogen. PKR^−/−^ mice infected with *T. gondii* exhibited higher parasite load and worsened histopathology in the eye and brain compared to wild-type controls. Susceptibility to toxoplasmosis was not due to defective expression of IFN-γ, TNF-α, NOS2 or IL-6 in the retina and brain, differences in IL-10 expression in these organs or to impaired induction of *T. gondii*-reactive T cells. While macrophages/microglia with defective PKR signaling exhibited unimpaired anti-*T. gondii* activity in response to IFN-γ/TNF-α, these cells were unable to kill the parasite in response to CD40 stimulation. The TRAF6 binding site of CD40, but not the TRAF2,3 binding sites, was required for PKR phosphorylation in response to CD40 ligation in macrophages. TRAF6 co-immunoprecipitated with PKR upon CD40 ligation. TRAF6-PKR interaction appeared to be indirect, since TRAF6 co-immunoprecipitated with TRAF2 and TRAF2 co-immunoprecipitated with PKR, and deficiency of TRAF2 inhibited TRAF6-PKR co-immunoprecipitation as well as PKR phosphorylation induced by CD40 ligation. PKR was required for stimulation of autophagy, accumulation the autophagy molecule LC3 around the parasite, vacuole-lysosomal fusion and killing of *T. gondii* in CD40-activated macrophages and microglia. Thus, our findings identified PKR as a mediator of anti-microbial activity and promoter of protection against disease caused by a non-viral pathogen, revealed that PKR is activated by CD40 via TRAF6 and TRAF2, and positioned PKR as a link between CD40-TRAF signaling and stimulation of the autophagy pathway.

## Author Summary

PKR was identified more than 30 years ago as an inhibitor of viral replication. It is unknown if PKR promotes protection against disease caused by non-viral pathogens. We addressed this question using *Toxoplasma gondii*, a major parasitic pathogen. *T. gondii* can cause cerebral and/or eye disease primarily in immunosuppressed patients and newborns. After infection with *T. gondii*, PKR-deficient mice exhibited high parasite loads in the eye and brain and were more susceptible to ocular and cerebral toxoplasmosis. Macrophages and microglia are important effectors of protection against *T. gondii*. These cells required PKR signaling to kill the parasite in response to stimulation via CD40, a molecule that promotes protection against ocular and cerebral toxoplasmosis. CD40 functioned only through its TRAF6 binding site to activate PKR, but this process was also dependent on TRAF2 where this molecule likely acted as an intermediary that promoted TRAF6-PKR association and PKR activation. PKR linked CD40-TRAF signaling to stimulation of the autophagy pathway and *T. gondii* killing. Our studies identified a previously unappreciated role of PKR as mediator of anti-microbial activity and promoter of resistance against disease caused by a non-viral pathogen, as well as provided new insight on the molecular link between CD40 and PKR.

## Introduction

PKR, also known and eukaryotic translation initiation factor 2-alpha kinase 2 (IEF2AK2) is a ubiquitously expressed serine-threonine kinase, constitutively present at low levels as inactive monomers in the cytoplasm of mammalian cells [1]–[3]. This kinase was discovered as a component of the interferon-inducible cellular antiviral defenses. PKR consists of a kinase domain (KD) and two tandem dsRNA binding domains (dsRBD) that regulate the kinase activity [2], [3]. Under resting conditions, dsRBD interact with the KD maintaining the molecule in a closed, inactive form [4], [5]. Binding of dsRNA to the dsRBD results in a conformational change that is believed to relieve the KD from the autoinhibitory effect of the dsRBD, allowing PKR to dimerize and autophosphorylate, thus becoming active [4], [5]. Activated PKR can then catalyze the phosphorylation of its best-characterized substrate, the subunit of eukaryotic initiation factor 2 (eIF-2α) leading to inhibition of protein synthesis [1]–[3].

The antiviral role of PKR has been well characterized. dsRNA produced during infection with RNA and DNA viruses causes PKR activation with resulting eIF2α phosphorylation and inhibition in viral protein translation. Moreover, *in vivo* studies revealed that PKR restricts replication of viruses such as vesicular stomatitis virus [6], [7], lymphocytic choriomeningitis virus [8] and Herpes simplex virus 2 (HSV-2) [9]. The importance of PKR in anti-viral immunity is emphasized by the fact that most animal viruses utilize various strategies to impair the action of PKR [3].

In addition to its role as a translational regulator, PKR is involved in signal transduction. PKR can signal to NF-κB, the signal transducer and activator of transcription (STAT)-1 and -3, IFN regulatory factor (IRF)-1, activating transcription factor (ATF)-3 and -4, p53 AP-1, Jun N-terminal protein kinase (JNK) and p38 [2], [3]. In addition, dsRNA, cytokines (IFN-γ, TNF-α, IL-1) [3], [10], [11], LPS [12], [13] and platelet-derived growth factor (PDGF) [14] can activate PKR. Moreover, the intracellular protein PKR-associated protein PACT (also called RAX in mice) can activate PKR in the absence of dsRNA [5], [15], [16].

While the role of PKR in antiviral immunity is well characterized, there is limited evidence for the involvement of this kinase during infections with non-viral pathogens. Induction of IL-6 and IL-12 p40 was defective in PKR^−/−^ fibroblasts exposed to LPS [13]. In addition, serum levels of these two cytokines were reduced in PKR^−/−^ mice challenged with LPS [13]. PKR promoted IL-6 and TNF-α production by mouse alveolar macrophages stimulated with TLR2 and TLR4 ligands [17]. Bacillus Calmette-Guerin (BCG) induced PKR-dependent IL-6, IL-10 and TNF-α production by human monocytes [18]. More recently, PKR activation was shown to enhance *in vitro* replication of the protozoan *Leishmania amazonensis* in human and mouse macrophages, an effect that appears to be mediated by PKR-dependent IL-10 production [19]. In addition, PKR^−/−^ mice exhibit improved *in vivo* control of *Mycobacterium tuberculosis* that is accompanied by increased apoptosis of infected macrophages and reduced macrophage production of IL-10 [20]. However, to our knowledge, it has not been reported whether PKR stimulates anti-microbial activity against a non-viral pathogen and enhances resistance against disease caused by such a pathogen.

*Toxoplasma gondii* is an obligate intracellular protozoan parasite that infects an estimated 30% of the human population worldwide. Tachyzoites, the invasive form of the parasite, penetrate host cells and reside within parasitophorous vacuoles that resist lysosomal fusion thereby avoiding eradication [21]. Tissue cysts are formed primarily in the brain and skeletal muscle during the chronic phase of infection and persist in the host for life. Infection with *T. gondii* can cause severe illness in children with congenital infection and in immunocompromised adults. Toxoplasmic encephalitis and ocular toxoplasmosis are two important manifestations of toxoplasmosis. Host protection against *T. gondii* infection is mediated primarily by T cell-mediated immunity [22]–[24]. IFN-γ, TNF-α and NOS2 are major mediators of resistance to both toxoplasmic encephalitis and ocular toxoplasmosis [25]–[31]. Using models of *T. gondii* infection we report that PKR contributes to protection against ocular and cerebral toxoplasmosis, triggers anti-microbial activity against this pathogen in macrophages and microglia and we identified molecular events involved in induction of this activity.

## Results

### PKR^−/−^ mice are susceptible to ocular and cerebral toxoplasmosis

**Figure 1:**
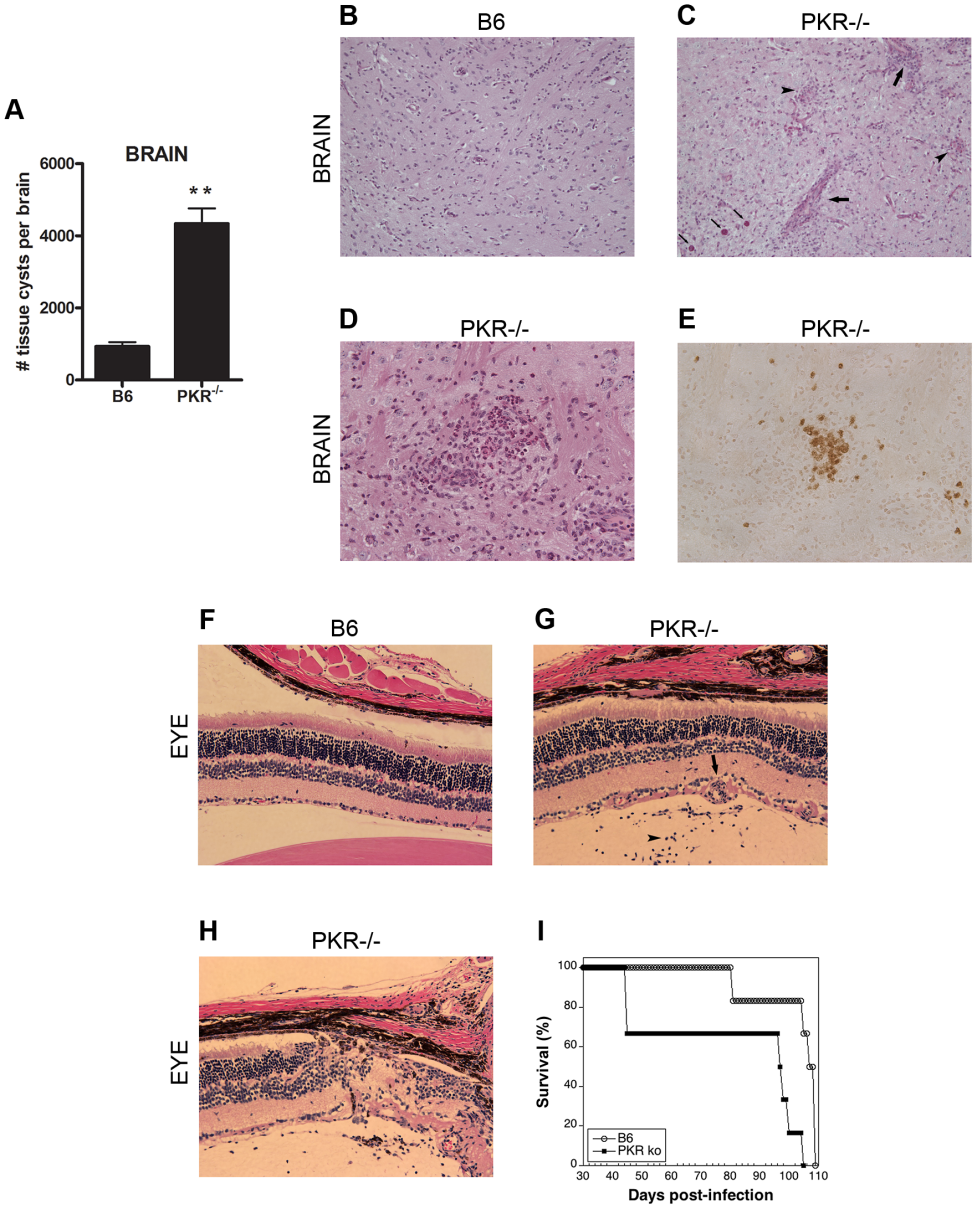
PKR^−/−^ mice are susceptible to cerebral and ocular toxoplasmosis. B6 and PKR^−/−^ mice were infected i.p. with 10 cysts of the ME49 strain of *T. gondii*. *A*. PKR^−/−^ mice exhibited a higher numbers of tissue cysts in the brain than B6 mice. Brains were homogenized and tissue cysts quantified by light microscopy. Results are shown as the mean ± SEM. *B*, Brains from infected B6 mice exhibited minimal histopathology. *C*, PKR^−/−^ mice show areas of perivascular inflammation (thick arrow), presence of microglial nodules (arrow head) and numerous tissue cysts (thin arrow). *B–C*, Periodic Acid Schiff-Hematoxylin (PASH); original magnification ×100. *D*, PKR^−/−^ mice also showed areas of acute inflammation (PASH; original magnification ×200). *E*, Immunohistochemistry using anti-*T. gondii* Ab revealed tachyzoites and *T. gondii* antigens (dark brown precipitate) associated with areas of acute inflammation. Staining after omission of primary Ab confirmed specificity of the immunohistochemical reaction. Images in *D* and *E* were taken from sections 5 µm apart. *F–H*, Compared to B6 mice (*F*), PKR^−/−^ mice (*G*) exhibit retinal, perivascular (thick arrow) and vitreal inflammation (arrow head). PKR^−/−^ mice (*H*) also exhibited disruption of retinal architecture, hypertrophy of retinal pigment epithelial (RPE) cell layer and retinal invasion by RPE cells (thin arrow). *F–H*, Hematoxylin and eosin (H&E); original magnification ×200. *I*, Survival curve of B6 and PKR^−/−^ mice infected with ME49. Results shown are representative of 3–4 independent experiments. **p<0.01.

To begin to explore the relevance of PKR during *T. gondii* infection, wild-type (B6) and PKR^−/−^ mice were infected with tissue cysts of the ME49 strain of *T. gondii*. Parasite load as assessed by qPCR for the *T. gondii* B1 gene was examined at different times throughout the first month post-infection. B6 and PKR^−/−^ mice had similar *T. gondii* parasite loads in the spleen, liver and lung at days 3, 7, 14 and 28 post-infection and both strains of mice were able to restrict the parasite load in these organs (Table 1). In contrast to peripheral organs, the parasite loads in the brain and eye were significantly higher in PKR^−/−^ compared to B6 mice, a difference that became more pronounced at day 28 post-infection (Table 1). At approximately this time, PKR^−/−^ mice exhibited piloerection and hunched posture. Brain homogenates of PKR^−/−^ mice collected at this time contained significantly greater numbers of tissue cysts than control B6 mice (Figure 1A). Histopathological examination at 4 weeks post-infection revealed that while there was minimal inflammation in brain sections from B6 mice (Figure 1B), brains of PKR^−/−^ mice exhibited a significant increase in perivascular inflammation, microglial nodules and presence of numerous tissue cysts (p<0.01) (Figure 1C). Areas of acute focal inflammation were noted (Figure 1D) in which tachyzoites and parasite antigens were detected (Figure 1E). Also, while B6 mice revealed minimal histopathological changes in the retina (Figure 1F), eyes from PKR^−/−^ mice had remarkable histopathological changes characterized by presence of inflammatory cells in the vitreous and retina including perivascular inflammation, distortion of the retinal architecture, hypertrophy of the retinal pigment epithelial cells (RPE) and invasion of the retina and vitreous by RPE (p<0.01) (Figure 1G,H). In addition, infected PKR^−/−^ mice exhibited increased mortality (Figure 1I). Thus, PKR promotes resistance against ocular and cerebral toxoplasmosis.

**Table 1:** *T. gondii* parasite load in B6 and PKR^−/−^ mice.

	Day 3		Day 7		Day 14		Day 28	
	B6	PKR^−/−^	B6	PKR^−/−^	B6	PKR^−/−^	B6	PKR^−/−^
Spleen	7,746	9,822	19,391	24,339	1,877	1,993	109	117
	±1,821	±3,292	±4,205	±4,293	±695	±314	±41.7	±17
Liver	735	626	23,009	22,330	761	1,179	Undetec	Undetec
	±120	±239	±578	±918	±77	±441		
Lung	Undetec	Undetec	112	162	408	687	44	Undetec
			±16	±47	±69	±160	±8	
Brain	Undetec	Undetec	115	246	2,359	8,023	42,300	166,360
			±16	±40*	±370	±2,050*	±4,887	±15785#
Eye	Undetec	Undetec	Undetec	41	131	342	290	1,647
				±8*	±26	±55*	±80	±274#

B6 and PKR^−/−^ mice were infected i.p. with 10 tissue cysts of the ME49 strain of *T. gondii*. Mice were euthanized at different times post-infection. Genomic DNA was isolated from various organs and levels of the *B1* gene of *T. gondii* were examined by quantitative PCR. A standard curve of DNA from known numbers of parasites per reaction was used to calculate the number of parasites per µg of genomic DNA isolated from organs. Results are shown as the mean ± SEM and are representative of 3 independent experiments (4–5 mice per group).

* p<0.05;

# p<0.01.

### PKR deficiency does not impair expression of IFN-γ, IL-12, TNF-α, IL-6, NOS2/nitric oxide, p47 GTPases, and does not affect expression of IL-10

**Figure 2:**
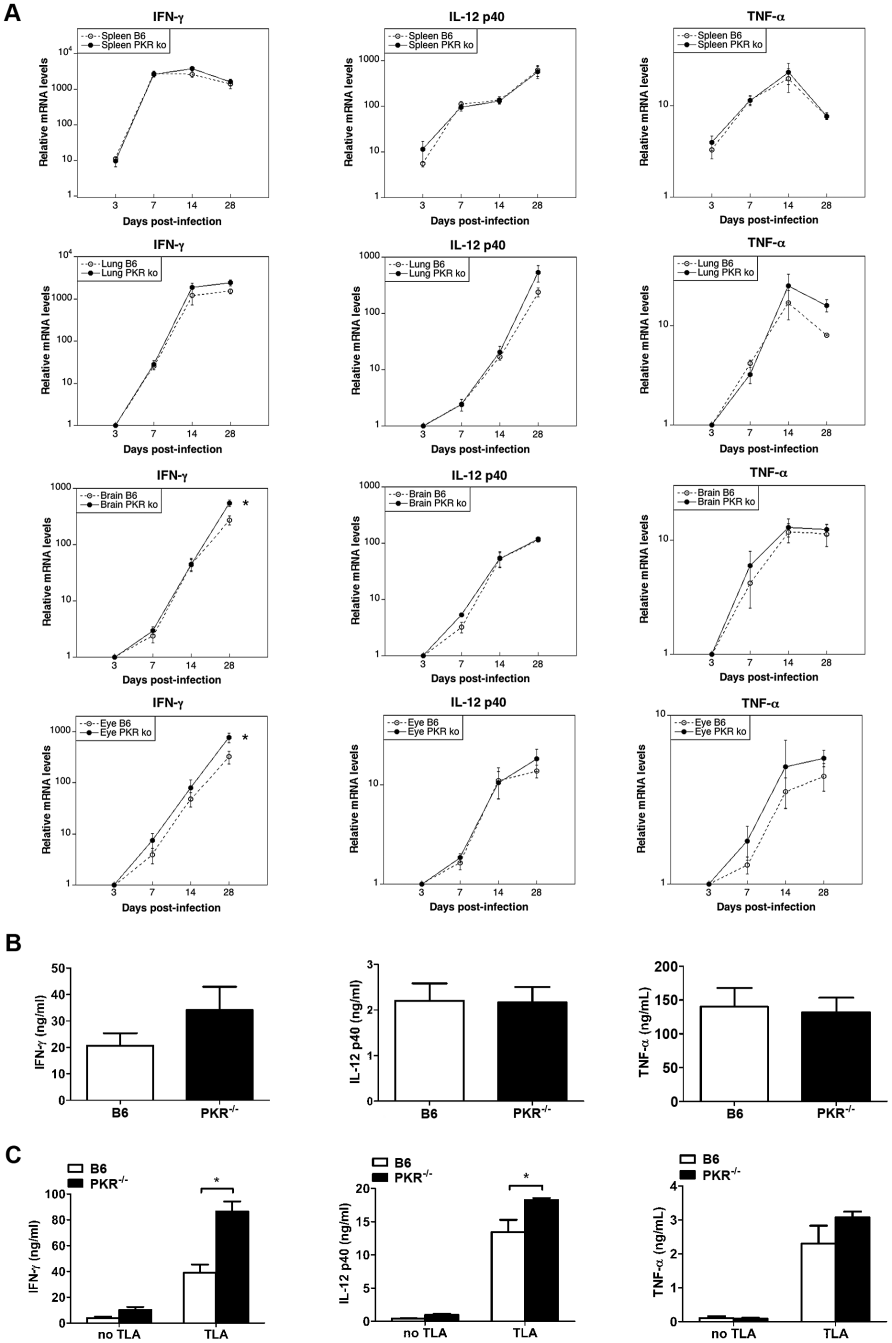
IFN-γ, IL-12 and TNF-α expression in *T. gondii*-infected B6 and PKR^−/−^ mice. B6 and PKR^−/−^ mice were infected i.p. with 10 cysts of the ME49 strain of *T. gondii*. Mice were euthanized at different times post-infection. *A*, Comparison of IFN-γ, IL-12 and TNF-α RNA levels between B6 and PKR^−/−^ mice. RNA was isolated from organs and levels of IFN-γ, IL-12 and TNF-α were examined by quantitative PCR and normalized against the levels of 18s rRNA. Increase in mRNA levels was calculated by comparing to uninfected organs. *B*, Concentrations of IFN-γ, IL-12 and TNF-α were measured by ELISA in sera collected at 7 days post-infection. *C*, Splenocytes were obtained at day 7 post-infection and incubated with or without TLA (10 µg/ml). IFN-γ IL-12 and TNF-α were measured by ELISA. Results are shown as the mean ± SEM and are representative of 3 independent experiments. *p<0.05.

**Figure 3:**
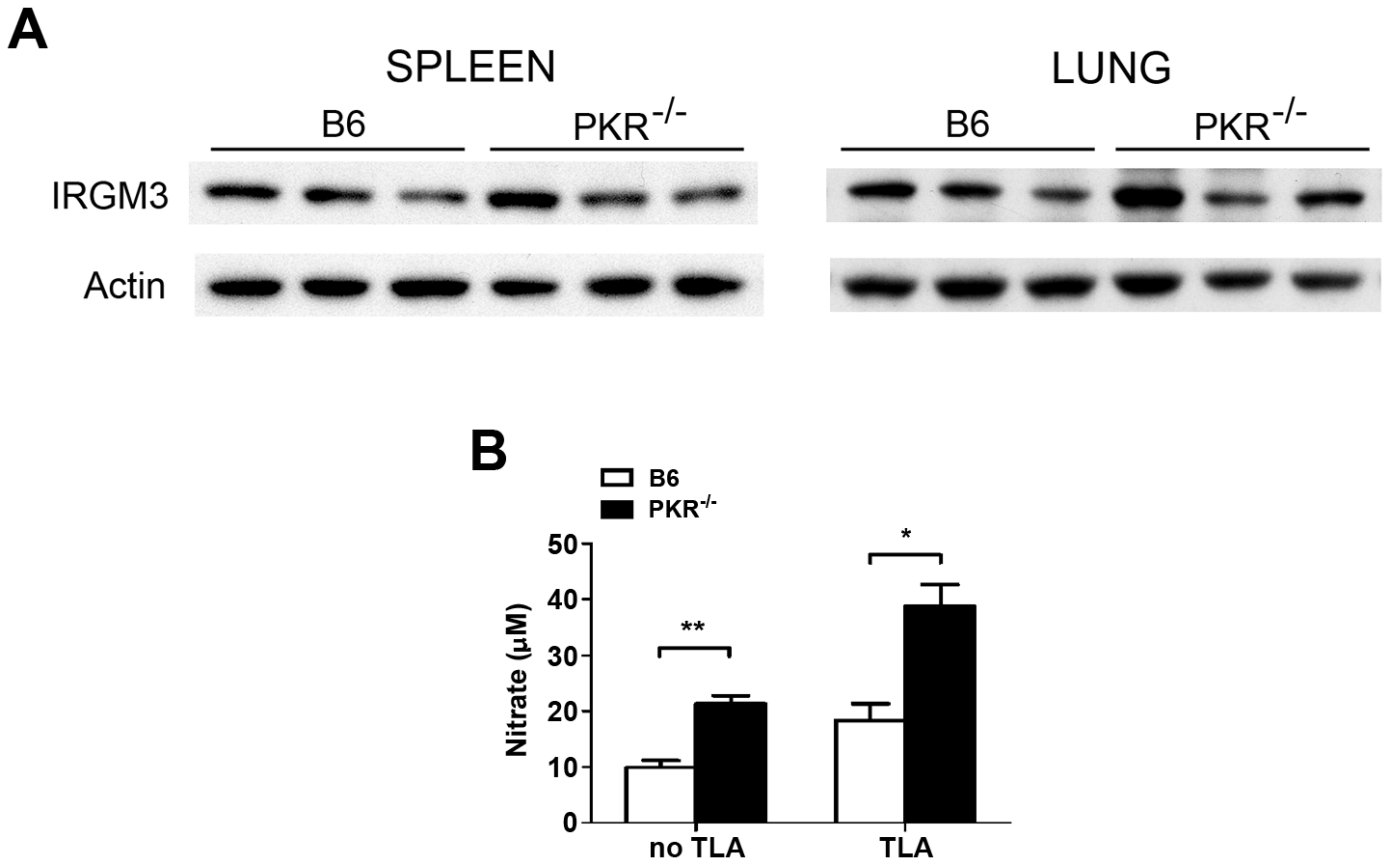
IRGM3 expression and nitric oxide production in *T. gondii*-infected B6 and PKR^−/−^ mice. B6 and PKR^−/−^ mice were infected i.p. with 10 cysts of the ME49 strain of *T. gondii*. *A*, Spleen and lung lysates were obtained at day 7 post-infection and used to measure IRMG3 levels by immunoblot. Each lane corresponds to a representative mouse. *B*, Splenocytes were obtained at day 7 post-infection and incubated with or without TLA. Nitric oxide was measured by Griess reaction in supernatants collected at 72 h. Results are shown as the mean ± SEM and are representative of 3 independent experiments. *p<0.05; **p<0.01.

PKR can enhance cytokine and NOS2 expression [13], [17], [18], [32]–[34]. IFN-γ is critical for control of *T. gondii*, and the production of this cytokine is dependent on IL-12 [25], [35]–[37]. TNF-α is also important for protection [30], [31]. mRNA levels of these cytokines were similar in infected B6 and PKR^−/−^ mice, with the exception of IFN-γ mRNA levels that were higher in the brain and eye of infected PKR^−/−^ mice (Figure 2A). Moreover, serum levels of IFN-γ, IL-12 and TNF-α were similar in infected B6 and PKR^−/−^ mice (Figure 2B). Splenocytes from PKR^−/−^ mice produced higher amounts of IFN-γ and IL-12 in response to *T. gondii* lysate antigens (Figure 2C). We also examined Immunity-related GTPases (IRG) expression and nitric oxide production, key effector molecules downstream of IFN-γ. Expression of IRGM3 was similar in the spleens and lungs from infected B6 and PKR^−/−^ mice (Figure 3A). Nitric oxide production by splenocytes incubated with *T. gondii* lysate antigens was higher in PKR^−/−^ mice (Figure 3B). Data shown on serum cytokine levels, cytokine and nitric oxide production by splenocytes and expression of IRGM3 are from samples obtained on day 7 post-infection. Samples collected on day 14 post-infection also revealed that the expression of these molecules was similar in B6 and PKR^−/−^ mice (not shown).

**Figure 4:**
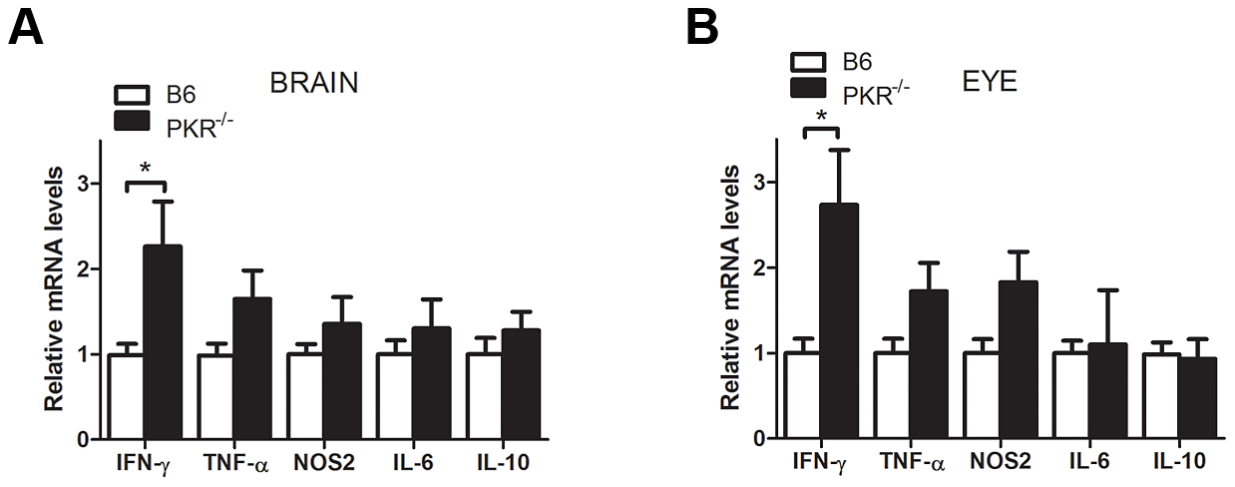
Cytokine and NOS2 expression in the brains and eyes of *T. gondii*-infected B6 and PKR^−/−^ mice. B6 and PKR^−/−^ mice were infected i.p. with 10 cysts of the ME49 strain of *T. gondii*. Mice were euthanized 4 weeks post-infection and RNA was isolated from brains (*A*) and eyes (*B*). Levels of IFN-γ, TNF-α, NOS2, IL-6 and IL-10 were examined by quantitative PCR and normalized against the levels of 18s rRNA. Results are shown as the mean ± SEM and are representative of 3–4 independent experiments. *p<0.05.

In addition to IFN-γ, TNF-α, cerebral and ocular mRNA levels of IL-6, NOS2 and IL-10 are increased in the brain and eye of mice infected with *T. gondii*, and these molecules promote protection against toxoplasmosis while in the case of IL-10, this cytokine modulates susceptibility to disease [25]–. We conducted a separate set of experiments to determine whether less effective parasite control in PKR^−/−^ mice might be due to defective expression of mediators of protection in the brain and eye or changes in IL-10 expression. As shown before, brains of infected PKR^−/−^ mice exhibited significantly higher mRNA levels of IFN-γ at 4 weeks post-infection (Figure 4A). The levels of TNF-α, NOS2 and IL-6 in PKR^−/−^ mice were comparable or even higher than those from B6 mice (Figure 4A). Assessment of expression of these molecules in the eye revealed similar results (Figure 4B). The mRNA levels of IL-10 in the brain and eye of PKR^−/−^ mice did not differ from B6 mice (Figure 4A–B). Taken together, PKR^−/−^ mice are more susceptible to *T. gondii* despite unimpaired expression of IFN-γ, TNF-α, NOS2 and IL-6 and lack of changes in IL-10 expression. In addition, it is unlikely that PKR deficiency promotes toxoplasmosis by impairing type I IFN signaling since we could not detect defective parasite control in the brains and eyes of IFN-α/βR^−/−^ mice (data not shown).

### PKR deficiency does not impair expansion of IFN-γ-producing T cells or anti-*T. gondii* IgG production

**Figure 5:**
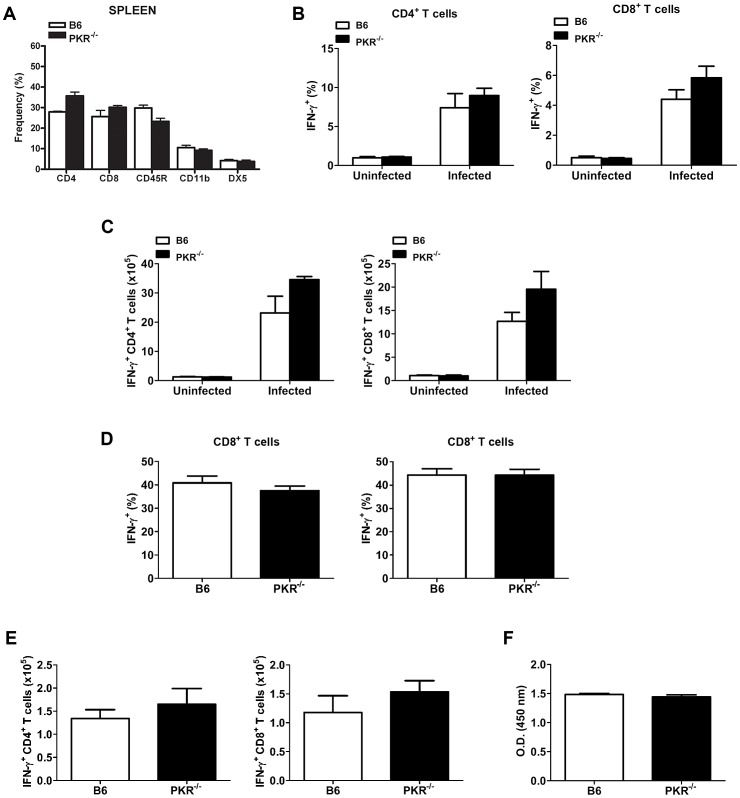
PKR^−/−^ mice exhibit unimpaired composition of lymphoid organs, expansion of IFN-γ^+^ T cells and anti-*T. gondii* IgG production. *A*, single cell suspensions of splenocytes from B6 and PKR^−/−^ mice were stained with anti-CD3, anti-CD4, anti-CD8, anti-CD11b, anti-CD45R, anti-CD49d (DX5) mAb followed by flow cytometric analysis. *B–F*, B6 and PKR^−/−^ mice were infected i.p. with 10 cysts of the ME49 strain of *T. gondii*. Splenocytes obtained at day 7 (*B, C*) and brain mononuclear cells obtained at 4 weeks post-infection (*D, E*) were cultured with anti-CD3 mAb and CD4^+^ and CD8^+^ cells were analyzed for intracellular IFN-γ by flow cytometry. *F*, Anti-*T. gondii* IgG detection in sera collected at 4 weeks post-infection. O.D. in sera from uninfected mice were ≤0.06. Results are shown as the mean ± SEM and are representative of 3 independent experiments.

PKR can control cellular proliferation, differentiation and apoptosis [41], [42]. Accordingly, we examined whether lack of PKR could perturb the cellular composition of a lymphoid organ. The frequencies of CD4^+^ T cells, CD8^+^ T cells, NK cells, monocytes and B lymphocytes were similar in the spleens of PKR^−/−^ and B6 mice confirming previous findings [43] (Figure 5A; p>0.05). Next, we examined the expansion of IFN-γ-producing T cells. Intracellular IFN-γ expression was examined after splenocytes were incubated with anti-CD3 mAb [44]. In contrast to splenocytes from uninfected animals, splenocytes from infected mice showed an expansion in the percentages of CD4^+^ T cells and CD8^+^ T cells that expressed IFN-γ as well as in the absolute numbers of IFN-γ-producing T cells (Figure 5B). The percentages and numbers of IFN-γ^+^ T cells were similar in B6 and PKR^−/−^ mice (Figure 5B). Similarly, the percentages of IFN-γ^+^ CD4^+^ and CD8^+^ T cells as well as the absolute numbers of these cells were comparable in brain mononuclear cells from infected B6 and PKR^−/−^ mice (Figure 5B) (p>0.2). These data indicate that lack of PKR does not affect the phenotypic composition of a lymphoid organ and does not impair the expansion of IFN-γ-producing T cells. PKR can modulate antibody production [45] and B cells play a protective role against *T. gondii* [46]. However, the levels of anti-*T. gondii* IgG antibodies as assessed by ELISA were similar in infected B6 and PKR^−/−^ mice. The anti-*T. gondii* IgG antibodies titers were 1∶25,600 in both groups of mice and the O.D. values (450 nm) at a serum dilution of 1∶200 were comparable (Figure 5C).

### PKR is required for CD40-induced *T. gondii* antimicrobial activity in macrophages and microglia

**Figure 6:**
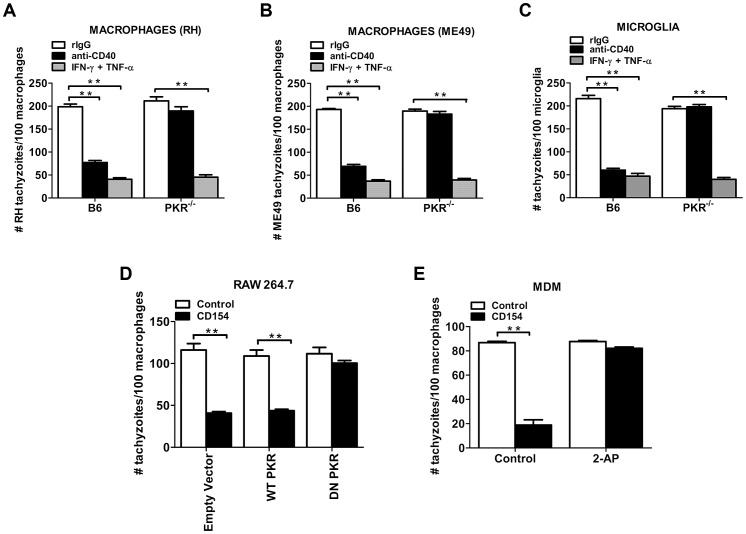
PKR is necessary for induction of antimicrobial activity in macrophages and microglia stimulated through CD40. Bone marrow-derived macrophages (*A, B*) or primary brain microglia (*C*) from B6 or PKR^−/−^ mice were incubated overnight with either control mAb, stimulatory anti-CD40 mAb or IFN-γ/TNF-α followed by challenge with type I (RH; *A, C*) or type II (ME49; *B*) strains of *T. gondii*. Monolayers were examined microscopically 24 h post-challenge. *D*, HmCD40-RAW 264.7 cells transfected with plasmids encoding WT-PKR, DN-PKR or empty plasmid were incubated with or without CD154 followed by challenge with *T. gondii*. Monolayers were examined microscopically 24 h post-challenge. *E*, Human MDM were incubated with or without CD154 in the presence or absence of 2-AP, followed by challenge with *T. gondii*. Monolayers were examined microscopically 48 h post-challenge. Results are shown as the mean ± SEM and are representative of 3 independent experiments. **p<0.01.

Macrophages and microglia are key effector cells that mediate resistance to *T. gondii* [26], [47], [48]. Thus, studies were performed to determine whether PKR is required for killing of *T. gondii* in these cells. Interestingly, treatment with IFN-γ plus TNF-α induced killing of *T. gondii* as efficiently in macrophages (Figure 6A–B) and microglia (Figure 6C) derived from PKR^−/−^ mice as in cells from B6 mice. These findings suggested a role for PKR in regulating another aspect of immune response to *T. gondii*. CD40 and its ligand CD154 are central for protection against ocular and cerebral toxoplasmosis [49], [50] and CD40 ligation activates macrophages and microglia to acquire anti-*T. gondii* activity [49], [51]–[54]. Accordingly, we assessed the role of PKR in the CD40-induced anti-*T. gondii* activity. Regardless of whether macrophages were infected with a type I (RH) or type II (ME49) strain of *T. gondii*, CD40 ligation caused a marked decrease in the number of tachyzoites in macrophages (Figure 6A–B). Anti-*T. gondii* activity was the same regardless of whether CD40 ligation took place before or after infection (not shown) [53]. CD40 ligation also caused anti-*T. gondii* activity in primary brain microglia (Figure 6C) from B6 mice. On the contrary, the parasite load did not decrease in CD40 stimulated macrophages and microglia from PKR^−/−^ mice. The lack of anti-*T. gondii* activity by PKR^−/−^ macrophages/microglia was not due to defective expression of CD40 by these cells as assessed by flow cytometric analysis (data not shown).

To further characterize the role of PKR in CD40-induced killing of *T. gondii* we utilized RAW 264.7 cells that express a chimera that consists of the extracellular domain of human CD40 and the intracytoplasmic domain of mouse CD40 (hmCD40) [53]. HmCD40-RAW 264.7 cells transiently transfected with plasmids encoding WT-PKR, DN-PKR (K296R) or empty plasmid were incubated with or without CD154 followed by challenge with *T. gondii*. While CD40 stimulation induced killing of *T. gondii* in cells transfected with either empty plasmid or wild-type PKR, anti-*T. gondii* activity was impaired in cells expressing DN-PKR (Figure 6D). Next, we determined whether PKR was relevant for controlling *T. gondii* in human macrophages. Prior to stimulation with CD154, human monocyte-derived macrophages (MDM) were treated with vehicle or 2-amino purine (2-AP), a pharmacological inhibitor of PKR kinase activity. Cells were then challenged with *T. gondii*. Stimulation with CD154 induced anti-*T. gondii* activity in MDM treated with vehicle alone. In contrast, 2-AP ablated anti-*T. gondii* activity in response to CD154 stimulation (Figure 6E). Taken together, these findings indicate that PKR is required for CD40-induced anti-*T. gondii* activity in macrophages and microglia but is dispensable for the IFN-γ/TNF-α arm of resistance to *T. gondii* in these cells.

### CD40 activates PKR independently of PACT, TNF-α, IL-1, IFN-α/β and IFN-γ

**Figure 7:**
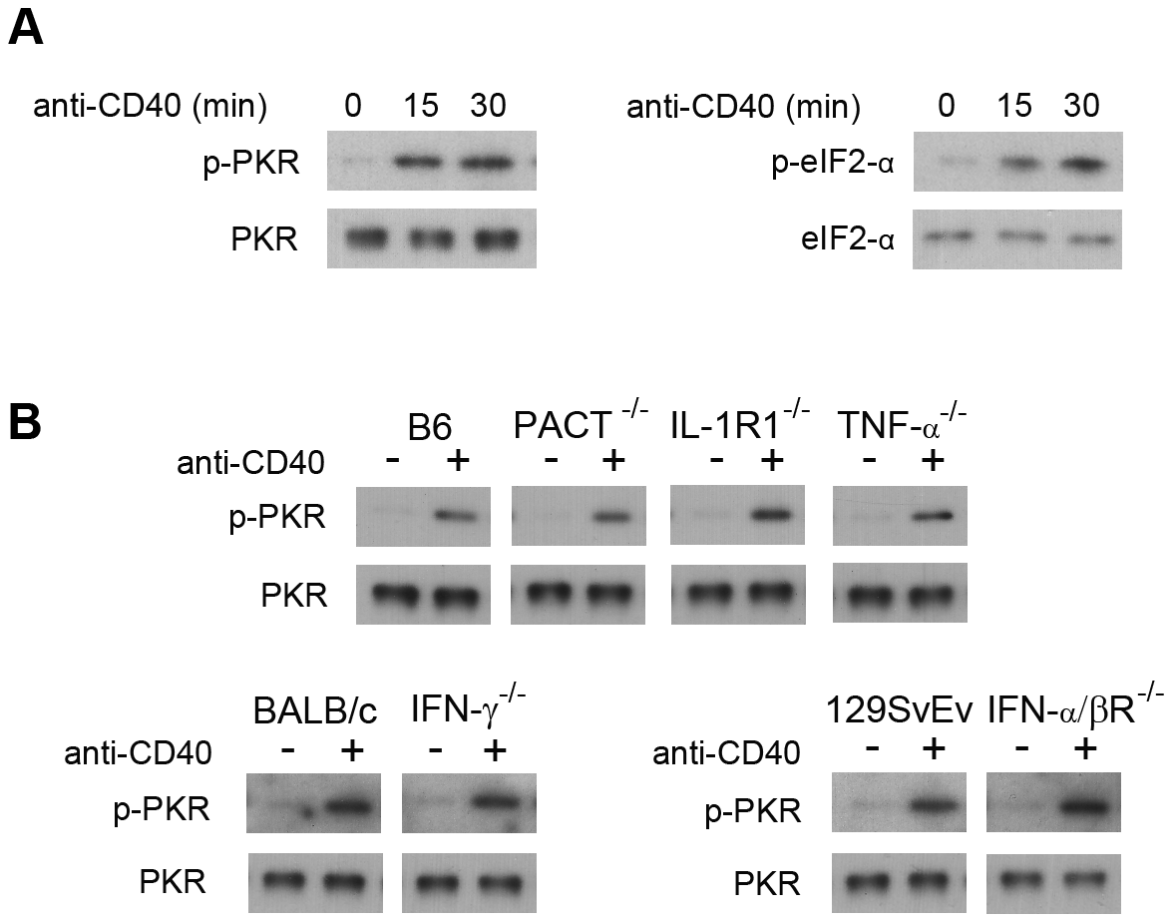
CD40 induces phosphorylation of PKR independent of IFN-γ, TNF-α, IL-1, IFN-α/β or PACT. *A*, Bone marrow-derived macrophages from B6 mice were treated with or without stimulatory anti-CD40 mAb. Cell lysates were used to probe for p-PKR, PKR, phospho-eIF2α and eIF2α by WB. *B*, Bone marrow-derived macrophages from wild-type, IFN-γ^−/−^, TNF-α^−/−^, IL-1R^−/−^, IFN-α/βR^−/−^ or PACT^−/−^ mice were treated with or without stimulatory anti-CD40 mAb. Cell lysates obtained after 30 min of *in vitro* stimulation were used to probe for p-PKR and PKR by WB. Results shown are representative of 3 independent experiments.

We determined whether CD40 ligation causes activation of PKR signaling in macrophages. Bone marrow derived macrophages from B6 mice incubated with a stimulatory anti-CD40 mAb exhibited PKR phosphorylation as assessed by immunoblot (Figure 7A). Similar results were obtained with monocyte-derived macrophages from humans (data not shown). In addition, CD40 stimulation caused phosphorylation of eIF2α, a signaling molecule classically activated by PKR (Figure 7A). PACT, TNF-α, IL-1 and IFNs can activate PKR [3], [5], [10], [11], [15], [16]. However, bone marrow-derived macrophages from PACT^−/−^, TNF-α^−/−^, IL-1R1^−/−^, IFN-α/βR^−/−^ and IFN-γ^−/−^ mice were not defective in phosphorylation of PKR in response to CD40 stimulation (Figure 7B). Thus, CD40 ligation induced phosphorylation of PKR that was independent of PACT, TNF-α, IL-1, IFN-α/β and IFN-γ.

### PKR associates with TRAF6 and TRAF2 in response to CD40 ligation

**Figure 8:**
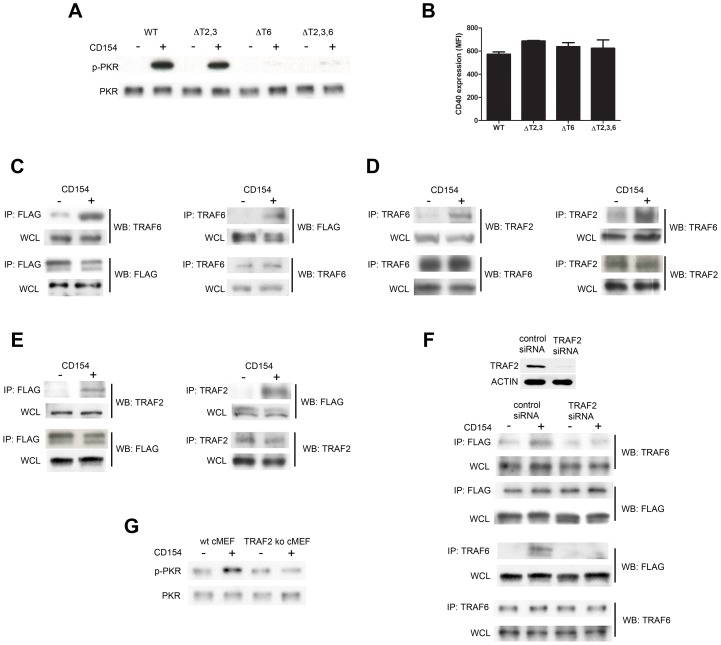
Role of CD40-TRAF binding sites and TRAFs in CD40-induced phosphorylation of PKR. *A*, RAW 264.7 cells that express WT hmCD40 or hmCD40 with mutations at the TRAF2/TRAF3 binding sites (ΔT2,3), TRAF6 binding site (ΔT6) or TRAF2/TRAF3 plus TRAF6 binding sites (ΔT2,3,6) were stimulated with CD154. Cell lysates obtained after 30 min of *in vitro* stimulation were used to probe for p-PKR and PKR by WB. *B*, Levels of CD40 in RAW 264.7 that express WT hmCD40 or hmCD40 ΔT2,3, ΔT6 or ΔT2,3,6. *C–E*, WT hmCD40-RAW 264.7 cells were transiently transfected with a plasmid encoding FLAG-tagged WT-PKR. Cells were then incubated with or without CD154 for 30 min. Lysates were immunoprecipitated by incubation with anti-FLAG, anti-TRAF6 or anti-TRAF2 antibodies and immunoblotted as indicated. *F*, WT hmCD40-RAW 264.7 cells were transfected with either control or TRAF2 siRNA followed by transfection with FLAG-tagged WT-PKR. Cells were incubated with CD154 and lysates were subjected to immunoprecipitation studies. *G*, wt MEF and TRAF2^−/−^ MEF that express hmCD40 were incubated with or without CD154. Cell lysates obtained after 30 min of *in vitro* stimulation were used to probe for p-PKR and PKR by WB. Results shown are representative of 3 independent experiments.

The cytoplasmic tail of CD40 lacks intrinsic catalytic activity and signals through its ability to recruit TNF receptor-associated factors (TRAFs) [55], [56]. Membrane-distal domains of CD40 directly bind TRAF2 and TRAF3 (TRAF3 inhibits CD40 signaling) whereas TRAF6 binds to a different membrane-proximal domain [55], [56]. CD40-induced toxoplasmacidal activity in macrophages is dependent exclusively on the TRAF6 binding site of CD40 [52], [54]. In order to examine the role of TRAF binding sites on PKR phosphorylation, RAW 264.7 cells that express WT hmCD40 or hmCD40 with mutations at the TRAF2,3 binding sites (ΔT2,3), TRAF6 binding site (ΔT6) or TRAF2,3 plus TRAF6 binding sites (ΔT2,3,6) [54] were stimulated with CD154. RAW 264.7 cells that express WT hmCD40 or hmCD40 with a mutation that disrupts binding to TRAF2,3 (ΔT2,3) exhibited unimpaired phosphorylation of PKR (Figure 8A). In contrast, phosphorylation of PKR was impaired in RAW 264.7 cells that express hmCD40 with a mutation that disrupts binding to TRAF6 (ΔT6) or mutations that disrupt binding to TRAF6 as well as TRAF2,3 (ΔT2,3,6) (Figure 8A). These results could not be explained by differences in the levels of CD40 expression (Figure 8B; p>0.1). Thus, PKR phosphorylation in response to CD40 ligation was dependent on the TRAF6 binding site.

PKR can associate with TRAF proteins including TRAF2, TRAF5 and TRAF6 [57]. We therefore hypothesized that TRAF6 links CD40 to PKR signaling. To test this hypothesis, FLAG-tagged PKR was transiently expressed in WT hmCD40-RAW 264.7 cells. Following stimulation with CD154, PKR immunoprecipitated with TRAF6 (Figure 8C). Interestingly, while PKR has been reported to contain binding motifs for TRAF2,3 [57], no TRAF6 binding motifs are identifiable in mouse PKR. However, TRAFs can form heterocomplexes [58], [59]. Thus, we examined whether CD40-induced association of TRAF6 and PKR is dependent on TRAF2. Following stimulation of WT hmCD40-RAW 264.7 cells with CD154, endogenous TRAF2 immunoprecipitated with endogenous TRAF6 (Figure 8D). In addition, TRAF2 immunoprecipitated with FLAG-tagged PKR (Figure 8E). Next, we examined the effects of TRAF2 knockdown to further determine the role of TRAF2 in the CD40-induced association between TRAF6 and PKR. Transfection of hmCD40-RAW 264.7 cells with TRAF2 siRNA effectively diminished TRAF2 protein levels (Figure 8F). TRAF2 knockdown diminished immunoprecipitation of TRAF6 and FLAG-tagged PKR in response to CD40 stimulation (Figure 8F). To further explore the role of TRAF2 in CD40-induced PKR phosphorylation wt MEF and TRAF2^−/−^ MEF that stably express hmCD40 were incubated with CD154. Whereas CD154 induced PKR phosphorylation in wt MEF, this effect was not observed in TRAF2^−/−^ MEF (Figure 8G). Taken together, while the TRAF2,3 binding sites of CD40 do not play an appreciable role in CD40-induced activation of PKR, the association of TRAF6 with PKR and the activation of PKR were dependent on TRAF2.

### PKR links CD40 to the autophagy pathway

**Figure 9:**
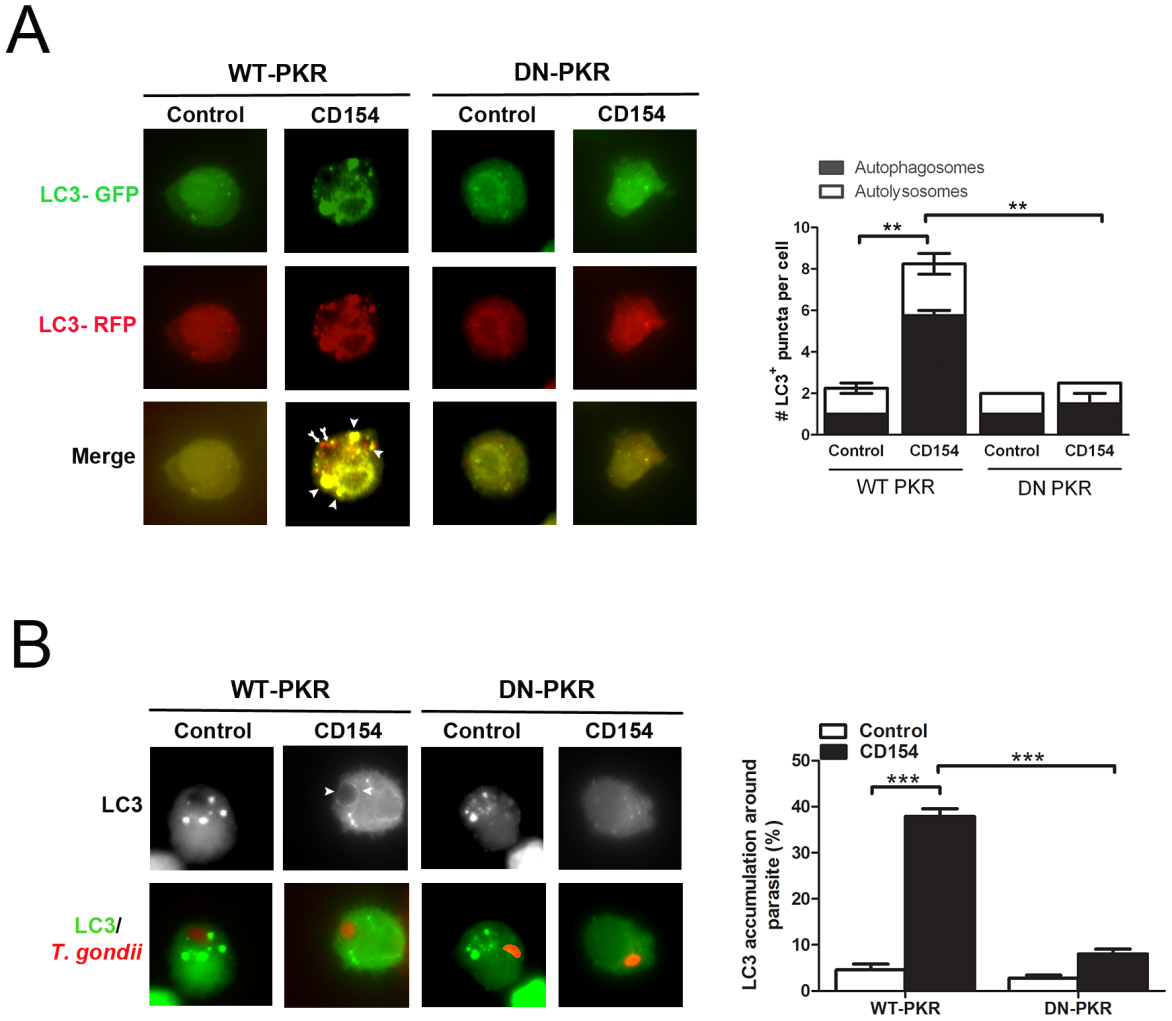
PKR mediates CD40-induced autophagy upregulation and CD40-induced recruitment of LC3 around *T. gondii*. *A*, HmCD40-RAW 264.7 cells were transiently transfected with either WT-PKR or DN-PKR. After 48 h, cells were transfected with tfLC3 followed by incubation with CD154 for 4 h. Monolayers were fixed and monitored by fluorescent microscopy for the number of autophagosomes (yellow) or autolysosomes (red). Autophagosomes and autolysosomes in CD154-stimulated cells that express WT-PKR are shown by arrowheads and arrows respectively. *B*, HmCD40-RAW 264.7 cells were transiently transfected with either WT-PKR or DN-PKR. After 48 h, cells were transfected with LC3-EGFP followed by incubation with CD154 overnight. Cells were challenged with transgenic RH tachyzoites that express RFP. 5 h post challenge, accumulation of LC3 around the parasite was examined by fluorescent microscopy (arrowheads). Results are shown as the mean ± SEM and are representative of 3 independent experiments. **p<0.01, ***p<0.001.

CD40 stimulation of *T. gondii*-infected macrophages and microglia leads to fusion of the parasitophorous vacuole with late endosomes/lysosomes, leading to lysosomal degradation and killing of the parasite [50], [53], [54]. This process is dependent on the autophagy machinery [50], [53], [54]. Autophagy is a conserved cellular homeostatic process whereby a double membrane autophagosome sequesters portions of the cytoplasm and damaged organelles and fuses with lysosomes culminating in the formation of an autolysosome and enzymatic degradation of its cargo [60]. The autophagy pathway is important for the control of *T. gondii* not only *in vitro* but also *in vivo* [50]. Accordingly, we examined whether PKR is required for stimulation of autophagy induced by CD40 stimulation of macrophages. We utilized a plasmid that encodes tandem fluorescent LC3 (tfLC3; RFP-GFP-tagged LC3) that enables to monitor both the presence of autophagosomes and the flux to autophagosome fusion with lysosomes (autolysosomes) [61]. CD40 stimulation of hmCD40 RAW 264.7 cells that expressed WT-PKR and were transfected with tfLC3 caused an increase in the percentages of cells with LC3^+^ autophagosomes and autolysosomes indicative of enhanced autophagy flux (Figure 9A). However, autophagy flux was markedly impaired in cells expressing DN-PKR (Figure 9A). Inhibition of PKR signaling did not affect the enhanced autophagy triggered by rapamycin (not shown). Next, we investigated whether PKR is required for recruitment of LC3 around the parasite. HmCD40-RAW 264.7 cells expressing LC3-EGFP plus either WT-PKR or DN-PKR were incubated with or without CD154 followed by challenge with transgenic *T. gondii* tachyzoites that express cytoplasmic RFP. CD40 stimulation of cells expressing WT-PKR resulted in accumulation of LC3 around the parasite (Figure 9B). In contrast, accumulation of LC3 was abrogated in cells expressing DN-PKR, pointing to the relevance of PKR in the CD40-induced recruitment of the autophagy protein LC3 around the parasite (Figure 9B).

**Figure 10:**
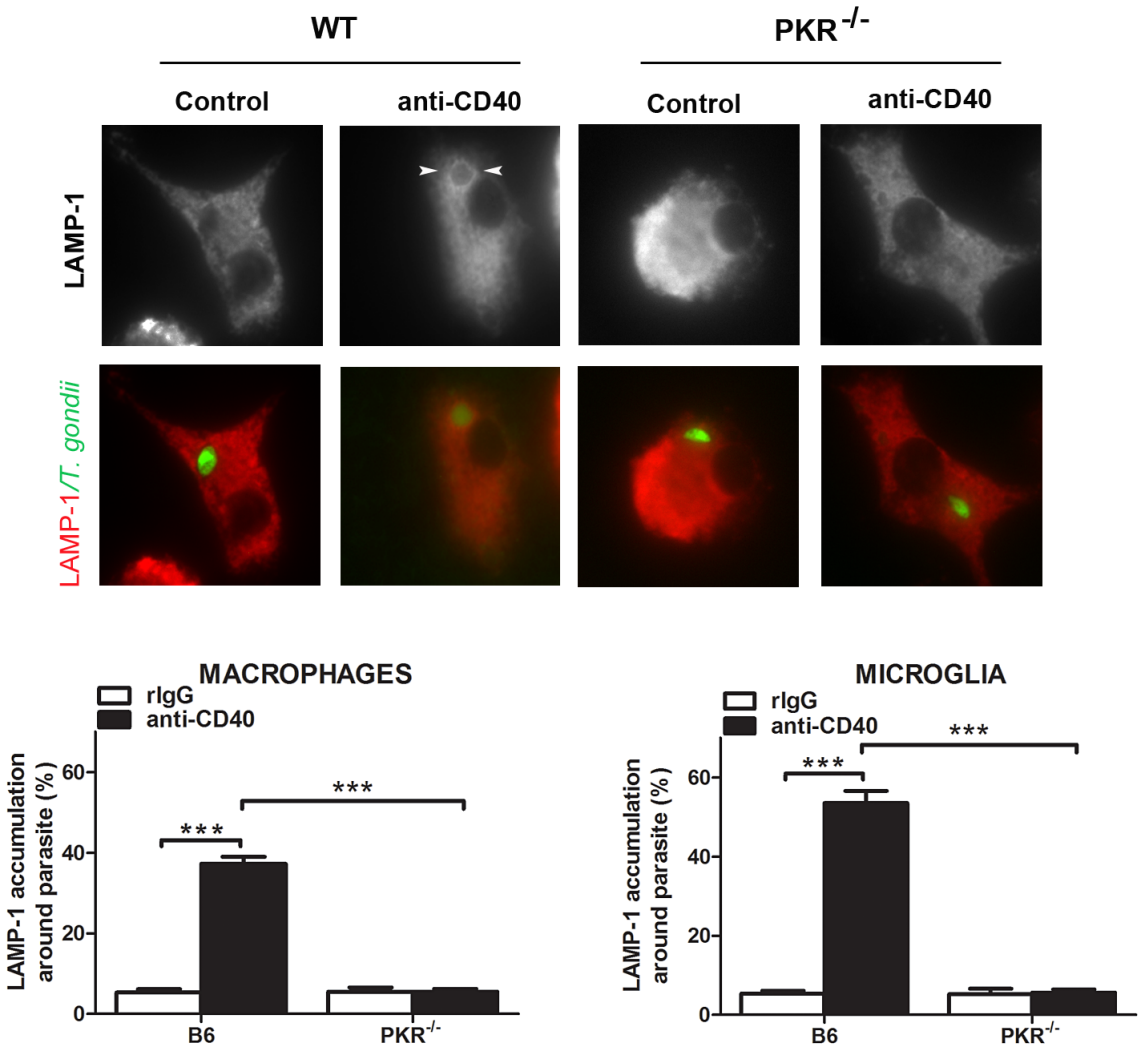
PKR mediates CD40-induced accumulation of LAMP-1 around *T. gondii*. Macrophages and microglia stimulated with anti-CD40 mAb were challenged with *T. gondii*-YFP. Monolayers were stained with anti-LAMP-1 mAb followed by Alexa Flour 568-conjugated secondary antibody. Accumulation of LAMP-1 around the parasite (arrowheads) was assessed by fluorescent microscopy 8 h post-challenge. Results are shown as the mean ± SEM and are representative of 3 independent experiments. ***p<0.001.

We previously showed that CD40 ligation results in fusion of late endosomes/lysosomes with the parasitophorous vacuole and killing of *T. gondii* [53]. Indeed, *T. gondii* infected bone marrow-derived macrophages or primary brain microglia from B6 mice exhibited accumulation of the late endosomal/lysosomal marker LAMP-1 around the parasite after CD40 ligation (Figure 10). In contrast, LAMP-1 accumulation was markedly impaired in cells derived from PKR^−/−^ mice (Figure 10). Taken together, PKR links CD40 to stimulation of the autophagy pathway, recruitment of the autophagy protein LC3 around *T. gondii* and vacuole-lysosomal fusion, the mechanism by which CD40 has been reported to mediate killing of *T. gondii* in macrophages and microglia [50], [53].

## Discussion

While the role of PKR in antiviral immunity has been extensively characterized, the involvement of PKR in mechanisms of protection against non-viral pathogens remains underexplored. We report herein that PKR^−/−^ mice exhibited increased parasite load and worsened histopathology in the eye and brain after infection with *T. gondii*. This was accompanied by impaired ability of macrophages and microglia to control the parasite in response to CD40-CD154 stimulation, molecules important for protection against the parasite in the eye and brain. Furthermore, we identified TRAF6 and TRAF2 as molecular links between CD40 and PKR activation. These findings indicate that PKR plays an important role in activation of mechanisms of protection against a non-viral pathogen.

In addition to induction of anti-viral activity in host cells via transcriptional inhibition, PKR has been reported to promote cytokine production, enhance nitric oxide production and appears to promote viral clearance mediated by CD8^+^ T cells [8], [13], [17], [18], [32]–[34]. We found no evidence of impaired expression of IFN-γ in PKR^−/−^ infected with *T. gondii*. Indeed, brain/eye IFN-γ mRNA levels were higher in these animals compared to controls. This phenomenon could be explained by the higher parasite loads detected in infected PKR^−/−^ mice and/or by a potential role of PKR in regulating transcription of IFN-γ mRNA [62]. Similar to studies of IFN-γ mRNA levels, the expression of IFN-γ^+^ CD4^+^ and CD8^+^ T cells was not impaired in PKR^−/−^ mice. This is relevant because CD4^+^ and CD8^+^ T cells are considered to mediate resistance against the parasite by producing IFN-γ [63]–[65]. Pertinent to our findings, the induction of LCMV-reactive CD8^+^ T cells is not defective in PKR^−/−^ mice [8]. In addition to IFN-γ production, CD8^+^ T cells exhibit cytotoxic activity against *T. gondii*-infected cells [66], [67]. However, it would appear unlikely that impaired induction of CD8^+^ T cell cytotoxic activity represents the major mechanism by which PKR promotes resistance to toxoplasmosis. PKR^−/−^ mice have increased tissue cysts in the brain and more severe encephalitis. In contrast, while CD8^+^ T cells that express perforin diminish the numbers of tissue cysts in the brain [68] and perforin^−/−^ mice infected with *T. gondii* exhibit higher tissue cyst numbers [65], these animals do not exhibit worse histopathology [65].

TNF-α, NOS2 and IL-6 are important mediators of protection against *T. gondii* in the brain and eye [26]–[31], [39]. However, we did not detect a defect in expression of these molecules in *T. gondii*-infected PKR^−/−^ mice. In addition to promoting cytokine production, PKR has been reported to mediate activation of NF-κB, MAPK and Akt in cells treated with IFN-γ, TNF-α and/or the combination of these cytokines [11], [69], [70]. Nevertheless, the induction of anti-*T. gondii* activity in response to IFN-γ/TNF-α was unimpaired in macrophages/microglia from PKR^−/−^ mice, a mouse macrophage line that expresses DN PKR and in human macrophages treated with 2-AP. Relevant to our results is the evidence that PKR plays a selective role in cytokine signaling since there are responses triggered by IFN-γ and TNF-α can occur independently of PKR [70]–[72]. In addition to being an IFN stimulated gene, PKR can promote type I IFN production [73]. However, our studies with IFN-α/βR^−/−^ mice argue against defective type I IFN signaling as explaining susceptibility to cerebral and ocular toxoplasmosis in PKR^−/−^ mice.

The CD40 - CD154 pathway activates macrophages/microglia to acquire anti-*T. gondii* activity [49]–[53]. Our studies revealed that in contrast to IFN-γ/TNF-α, CD40 stimulation requires PKR for induction of anti-*T. gondii* activity in macrophages/microglia. Relevant to this differential role of PKR is the evidence that CD40 does not require IFN-γ to activate macrophages/microglia to kill the parasite [50], [52]. However, despite the latter findings, the CD40-CD154 pathway functions in synergy with IFN-γ to enhance resistance to *T. gondii in vitro* and also likely *in vivo* [50], [51], [74]. The role of PKR in mediating anti-*T. gondii* activity induced by CD40 ligation may provide an explanation for susceptibility to ocular and cerebral toxoplasmosis in PKR^−/−^ mice since macrophages/microglia are considered to be key effectors of protection against the parasite in neural tissue and CD40^−/−^ and CD154^−/−^ mice are susceptible to these forms of the disease [26], [47]–[50]. Ligands for CD40 in the eye and brain of *T. gondii* infected mice may include not only infiltrating T cells, but potentially non-T cells that acquire CD154 expression as a result of cytokine stimulation as well as HSP70, a molecule upregulated in *T. gondii*-infected cells [75]–[77].

CD40 ligation induces phosphorylation of PKR in macrophages. PKR mediates signaling induced by TNF-α, IFN-γ, IFN-α/β and IL-1β [3], [10], [11], [69]–[71]. Moreover, CD40 enhances production of TNF-α, IL-1β and likely IFN-α [78], [79]. However, studies using macrophages from TNF-α^−/−^, IL-1R1^−/−,^ IFN-γ^−/−^ and IFN-α/βR^−/−^ mice indicate that PKR activation induced by CD40 is unlikely to be mediated by autocrine secretion of these cytokines. PACT/RAX is considered to be the intracellular mediator that links a wide variety of cellular stresses to PKR activation [15], [16]. Our studies also indicate that PKR phosphorylation induced by CD40 was independent of PACT/RAX. Moreover, CD40-induced killing of *T. gondii* was unimpaired in bone marrow derived macrophages from PACT^−/−^ mice (Portillo et al; unpublished observations). These findings indicate that there is a mechanism distinct from cytokine secretion and PACT activation by which CD40 induces PKR phosphorylation.

The cytoplasmic tail of CD40 lacks intrinsic kinase activity and therefore signals through recruitment of adaptor proteins. TRAFs are central mediators of CD40 signaling. Our data herein show that the TRAF6 binding site in the intra-cytoplasmic tail of CD40 is required for phosphorylation of PKR. These results are consistent with the pivotal role of the TRAF6 binding site in the induction of anti-*T. gondii* activity in CD40-activated macrophages [52]. TRAF recruitment to cytoplasmic domains of receptor molecules can lead to assembly of larger signaling complexes. Indeed, in our immunoprecipitation experiments, TRAF6 was observed to interact with PKR in response to CD40 ligation. While TRAFs are proposed to act downstream of human PKR and mediate NF-κB activation [57], we are unaware of studies on TRAFs as upstream regulators of PKR. Human PKR can interact with TRAFs in HeLa and 293T cells infected with vaccinia virus expressing PKR, transfected with PKR- or TRAF-encoding plasmids or treated with IFN-α/β [57]. Two putative TRAF-binding sites exist in human PKR: one in the dsRBD II subdomain (TKQE) and another in the KD (PEQIS) [57]. Both sites have been reported to interact with TRAF2 [57]. However, whereas the TRAF interacting site in dsRBD II is preserved in mouse PKR, the KD site in mice exhibits an altered motif (PEQLF). The presence of phenylalanine in the C-terminus of this motif is predicted to ablate recruitment of TRAF2 [80]. Thus, TRAF-PKR interaction in mice would most likely occur at the level of the dsRBD II subdomain since no other putative TRAF binding domain is apparent. Binding of TRAF proteins to dsRBD II subdomain could potentially result in an open (active) PKR conformation given that the dsRBD – KD interaction keeps PKR in a closed (inactive) form. In this regard, PACT is believed to activate PKR by binding to the site in KD that interacts with dsRBD II subdomain resulting in allosteric changes in PKR and an open conformation [5].

Of importance to our studies, no apparent TRAF6 binding site is detected in mouse PKR [81]. This suggested the possibility that the interaction between TRAF6 and PKR is indirect via TRAF2. Indeed, our studies indicate that upon CD40 ligation there is TRAF6-TRAF2 and TRAF2-PKR interaction. Moreover, TRAF2 deficiency impairs CD40-induced TRAF6-PKR association and PKR phosphorylation even though the TRAF2,3 binding site of CD40 plays no appreciable role in PKR activation. Of relevance to our studies, TRAFs can form heterocomplexes through their TRAF domains. TRAF3 can interact with TRAF5 [58], while TRAF2 may recruit TRAF6 to the cytoplasmic tail of CD40 in non-hematopoietic cells [59]. It has recently been reported that CD40 appears to induce PKR phosphorylation in B cells although the molecular mechanisms responsible for this effects were not elucidated [45]. It remains to be determined if CD40-induced TRAF6-TRAF2 signaling may be responsible for PKR activation in these cells.

Autophagy can act as an anti-microbial mechanism against several pathogens including *T. gondii* [82]. CD40 stimulation of macrophages and microglia results in killing of *T. gondii* dependent on the autophagy proteins Beclin 1 and Atg 7 [50], [53], [54]. PKR signaling has been implicated in regulation of the autophagy pathway. PKR controls autophagy triggered by starvation [83]. In addition, PKR can modulate autophagy in response to virus infection. Using long-lived protein degradation as an indicator of autophagy activity, PKR was shown to promote autophagy in response to HSV-1 infection, a process that is inhibited by the HSV-1 neurovirulence factor ICP34.5, a protein that antagonizes autophagy by binding to Beclin 1 [84]. While wild-type mice infected with a mutant HSV-1 that lacks the neurovirulence factor ICP34.5 do not develop encephalitis, PKR^−/−^ mice are susceptible to this disease suggesting a role of autophagy in protection against HSV-1 encephalitis [85]. In this study, we identified PKR as a molecular link between CD40 and the autophagy pathway since PKR is required for the CD40-induced autophagy flux, the accumulation of the autophagy protein LC3 around *T. gondii*, vacuole-lysosomal fusion and killing of the parasite. PKR appears to promote autophagy induced by selective stimuli since we have not observed a significant role for PKR in autophagy enhanced by rapamycin. While CD40 likely functions in synergy with IFN-γ to enhance resistance to *T. gondii in vivo*, the role of PKR as a link between CD40 and killing of *T. gondii* via the autophagy pathway may contribute to increase the resistance to ocular and cerebral toxoplasmosis. *T. gondii* can impair the effects of IFN-γ by manipulating cell signaling in host cells and by inducing *in vivo* production of cytokines that can have antagonistic activity against IFN-γ [86]. The presence of a process such as CD40-induced autophagy that can occur in the absence of IFN-γ and that is likely under different regulation than IFN-γ-induced anti-*T. gondii* activity may explain a role in enhancing protection in the setting of parasite-induced impairment of optimal IFN-γ signaling.

CD40-TRAF6 signaling triggers macrophage anti-*T. gondii* activity that is dependent on autocrine production of TNF-α [51]. However, TNF-α alone is not sufficient to induce anti-*T. gondii* activity in macrophages [52] indicating that induction of this activity requires synergy between CD40-TRAF6-induced TNF-α production and additional signals downstream of TRAF6 [53], [54]. Our studies indicate that PKR is unlikely to be activated by autocrine TNF-α production. However, we cannot rule out a potential role of PKR in mediating signals downstream of TNF-α.

While PKR can regulate various signaling pathways and modulates many cellular responses, one of the best-characterized roles of PKR is that of restricting viral replication. This study uncovered a role for PKR in inducing anti-microbial activity against a non-viral pathogen and enhancing protection against disease caused by this organism. In addition, this study reports a molecular link between CD40 (an important mediator of resistance to pathogens), TRAF2, TRAF6, PKR and activation of anti-microbial activity in macrophages/microglia.

## Materials and Methods

### Ethics statement

This study was carried out in strict accordance with the recommendations in the Guide for the Care and Use of Laboratory Animals of the National Institutes of Health. The protocol was approved by the Institutional Animal Care and Use Committee of Case Western Reserve University School of Medicine (Protocol Number 2009-0095).

### Animals and parasites

C57BL/6 (B6) mice were purchased from Jackson Laboratories. B6 and PKR^−/−^ mice (B6 background; lacking the majority of the amino-terminal dsRBD [87]) were maintained at the Animal Resource Center (Case Western Reserve University) and the Biological Resources Unit (Lerner Research Institute, Cleveland Clinic). Female mice were 8–12 weeks old when used for the studies (4–8 mice per group). Mice were infected intraperitoneally (i.p.) with 10 cysts of the ME49 strain of *T. gondii* (gift from Dr. George Yap, University of Medicine and Dentistry of New Jersey). In addition to B6 and PKR^−/−^ mice, PACT^−/−^ (Lerner Research Institute, Cleveland Clinic), IFN-γ^−/−^, TNF-α^−/−^, IL-1R1^−/−^, BALB/c (all from Jackson Laboratories), IFN-α/βR^−/−^ (gift from Dr. Clifford Harding, Case Western Reserve University) and 129SvEv mice (Taconic Farms) were used to obtain bone marrow-derived macrophages. Tachyzoites of the RH and PTG-ME49 strain were maintained in human foreskin fibroblasts. Transgenic parasites expressing cytoplasmic yellow fluorescent protein (YFP) or cytoplasmic DsRed (RFP) have been described [88], [89].

### Histopathology

Animals were anesthetized, perfused with PBS and euthanized. Four 5 µM sections of different areas of the brains and eyes were stained with periodic acid Schiff hematoxylin (PASH) or hematoxylin and eosin stain respectively. Histopathologic changes were scored following previously described criteria [26], [27]. In addition, immunohistochemistry to detect *T. gondii* parasites and antigens was performed as described [31], [38].

### Real-time quantitative PCR

Total RNA was isolated from brains and eyes using RNeasy kit (QIAGEN) according to the manufacturer's protocol. RNA (0.5 µg) was treated with DNase (Ambion) and reverse transcribed to cDNA with Super-Script III reverse transcriptase (Invitrogen) and oligo(dT)_12–18_ primers (Invitrogen). cDNA (2.5 µl) was used as template for quantitative RT-PCR using SYBR GREEN PCR mix (Applied Biosysytems) and 20 pM of primer in 50 µl. Primer sequences for IFN-γ [90], TNF-α [90], NOS2 [91], IL-6 [92], IL-10 [90], IL-12 p40 [93] and 18S rRNA [94] were previously described. Gene expression was assessed using 7300 Real Time PCR System (Applied Biosystems). Each sample was run in duplicate and normalized to the content of 18S rRNA [50]. Genomic DNA was isolated from organs using DNeasy kit (QIAGEN) and subjected to quantitative RT-PCR using SYBR GREEN PCR mix. A standard curve of DNA from 1 to 10^5^ ME49 tachyzoites per reaction was used to quantitate parasite load. Each sample was run in triplicate [50].

### Flow cytometry

Splenocytes were stained with anti-CD3, anti-CD4, anti-CD8, anti-CD40, anti-CD45R, anti-CD49d (DX5), anti-CD11b or isotype control mAb (all from eBiosciences). Cells were fixed with 1% paraformaldehyde and analyzed by use of an LSR II flow cytometer (BD Biosciences). Expression of intracellular cytokines was assessed in splenocytes and brain mononuclear cells. The latter cells were isolated as previously described [50]. Splenocytes obtained at 7 and 14 d post-infection as well as brain mononuclear cells obtained at 28 d post-infection were incubated with or without anti-CD3 plus Brefeldin A (10 µg/ml; eBiosciences) as described [44]. Cells were first stained with anti-CD3, anti-CD4 or anti-CD8. Cells were permeabilized using IntraPrep permeabilization reagent (Counter-Immunotech), following the manufacturer's protocol. Cells were then stained with anti-IFN-γ or anti-IL-4 mAb (eBiosciences). After fixation with 1% paraformaldehyde, cells were analyzed by use of an LSR II flow cytometer (BD Biosciences).

### Macrophages and microglia *in vitro* infection with *T. gondii*

Primary bone marrow-derived macrophages and brain microglia were obtained from control or PKR^−/−^ mice as described [50], [95]. Prior to infection, these cells were incubated overnight with isotype control or stimulatory anti-CD40 mAb (1C10; 10 µg/ml) or with IFN-γ (100 U/ml; PeproTech) plus TNF-α (250 pg/ml; PeproTech). RAW 264.7 cells stably transfected with linearized pRSV.5 plasmid encoding a chimera of the extracellular domain of human CD40 and the intracytoplasmic domain of mouse CD40 (hmCD40-RAW 264.7) were previously described [53]. HmCD40-RAW 264.7 cells were treated with or without CD154 (3 µg/ml; gift from W. Fanslow, Amgen, Thousand Oaks, California, USA) prior to infection with *T. gondii*. Human monocyte-derived macrophages were obtained as described [51] and were incubated with the PKR inhibitor, 2-aminopurine (2-AP; 2 mM; Sigma) or vehicle for 30 minutes followed by incubation with or without CD154. Tachyzoites of the RH or PTG-ME49 strains of *T. gondii* were used to infect monolayers as described [95]. Monolayers were fixed with Diff-Quick (Dade Diagnostics) and the number of tachyzoites per 100 cells was determined by light microscopy by counting at least 200 cells per monolayer as previously described [95].

### Transfections and retroviral vectors

HmCD40-RAW 264.7 cells were transiently transfected with a plasmid that encodes either FLAG-tagged wild-type (WT)-PKR, dominant negative (DN)-PKR (K296R), empty plasmid (gifts from Bill Sudgen, University of Wisconsin), TRAF2 siRNA [96] or control siRNA (Dharmacon) using an Amaxa Nucleofector (Amaxa) according to the manufacturer's protocol. For assessment of autophagy, hmCD40-RAW 264.7 cells were transfected with LC3-EGFP or a plasmid encoding tandem monomeric RFP-GFP-tagged LC3 (tfLC3) [61] (gifts from T. Yoshimori, National Institute for Basic Biology, Okazaki, Japan). Parent RAW 264.7 cells (>96% CD40^−^) were transduced with previously described EGFP-encoding MIEG3 retroviral vectors that encode WT hmCD40 or hmCD40 with mutations at the TRAF2,3 binding site (ΔT2,3), TRAF6 binding site (ΔT6) or TRAF2,3 plus TRAF6 binding sites (ΔT2,3,6) [54]. Briefly, parent RAW 264.7 cells were incubated with retroviral supernatants for 8 h in the presence of polybrene (8 µg/ml; Sigma Chemical). EGFP^+^ cells were sorted by FACS after 4 days. Mouse embryonal fibroblasts from wt and TRAF2^−/−^ mice (gifts from Hiroyasu Nakano) were also transduced with the retroviral vector that encodes wt hmCD40.

### Fluorescent microscopy

To assess autophagy flux, hmCD40-RAW 264.7 cells expressing tfLC3 plus either WT-PKR or DN-PKR were cultured with or without CD154 for 4 hr and fixed with 4% paraformaldehyde. Slides were mounted with Flouromount G (Southern Biotech) and analyzed by fluorescent microscopy for distinct LC3 positive structures that measure at least 1 µm in diameter [53]. For assessment of accumulation of LC3 around the parasite, hmCD40-RAW 264.7 cells expressing LC3-EGFP plus either WT-PKR or DN-PKR were cultured with or without CD154 overnight prior to challenge. Monolayers were infected with RH *T. gondii* that express cytoplasmic RFP. Five hr post challenge, monolayers were fixed with paraformaldehyde and assessed for LC3-EGFP accumulation around *T. gondii* as described [53]. For assessment of LAMP-1 accumulation around the parasite, macrophages or microglia were cultured with isotype control or stimulatory anti-CD40 mAb overnight prior to challenge with transgenic RH *T. gondii* that express cytoplasmic YFP. Monolayers were incubated with LAMP-1 antibodies (gift from Dr. Clifford Harding, Case Western Reserve University) followed by incubation with Alexa flour 568 conjugated secondary antibody (Jackson ImmunoResearch) and accumulation of LAMP-1 around the parasite was assessed 8 hr post infection as described [53].

### Immunoblotting and immunoprecipitation

Cells and organs (spleen, lung) were lysed in buffer supplemented with protease and phosphatase inhibitors (Cell Signaling). Equal amounts of protein were subjected to SDS-PAGE and transferred to a PVDF membrane. Membranes were probed with antibodies to total PKR or phospho PKR (Thr 451) (Santa Cruz Biotechnology), IRGM3 (Abcam, Cambridge, MA) or actin (Santa Cruz Biotechnology) followed by incubation with corresponding secondary Ab conjugated to horseradish peroxidase (Santa Cruz Biotechnologies). Bands were visualized by using a chemilluminescent kit (Pierce Bioscience). For immunoprecipitation, hmCD40-RAW 264.7 cells were transfected with a plasmid encoding FLAG-tagged WT-PKR or remained untransfected, and after 48 h, cells were incubated with or without CD154 for 30 min. In certain experiments, hmCD40-RAW 264.7 cells were transfected with either control or TRAF2 siRNA followed by transfection with FLAG-tagged WT-PKR after 24 h. Lysates were immunoprecipitated by incubation with anti-FLAG antibody (Sigma), anti-TRAF2 C20 antibody (Santa Cruz Biotechnology) or anti-TRAF6 D-10 antibody (Santa Cruz Biotechnology) overnight at 4°C. Protein complexes were then captured by incubation with 50 µl of protein G beads (Sigma) for 2 hr at 4°C and then washed with wash buffer supplemented with protease and phosphatase inhibitors. The beads were resuspended in 35 µl of sample buffer and boiled. Lysate from the immunoprecipitation was immunoblotted for TRAF2, TRAF6 or FLAG.

### Cytokine ELISA and nitric oxide measurement

Splenocytes (2×10^6^/ml) were incubated with or without TLA (10 µg/ml). Supernatants were collected at 24 h and used to determine concentrations of IL-12p 40 and TNF-α while supernatants collected at 72 h were used to measure IFN-γ (eBiosciences, San Diego, CA). Concentrations of nitric oxide were assessed in 72 h supernatants using Griess reaction (Promega Corporation, Madison, WI). Data are expressed as µM of nitrite.

### Statistical analyses

Statistical significance was assessed by 2-tailed student's *t* test and Analysis of Variance. Histopathologic changes were analyzed using Mann-Whitney *U* test. Differences were considered statistically significant when *p* was <0.05.
